# Competing Orders and Anomalies

**DOI:** 10.1038/srep31051

**Published:** 2016-08-08

**Authors:** Eun-Gook Moon

**Affiliations:** 1 Kadanoff Center for Theoretical Physics, University of Chicago, Chicago, IL 60637, USA; 2 Department of Physics, Korea Advanced Institute of Science and Technology, Daejeon 305-701, Korea

## Abstract

A conservation law is one of the most fundamental properties in nature, but a certain class of conservation “laws” could be spoiled by intrinsic quantum mechanical effects, so-called quantum anomalies. Profound properties of the anomalies have deepened our understanding in quantum many body systems. Here, we investigate quantum anomaly effects in quantum phase transitions between competing orders and striking consequences of their presence. We explicitly calculate topological nature of anomalies of non-linear sigma models (NLSMs) with the Wess-Zumino-Witten (WZW) terms. The non-perturbative nature is directly related with the ’t Hooft anomaly matching condition: anomalies are conserved in renormalization group flow. By applying the matching condition, we show massless excitations are enforced by the anomalies in a whole phase diagram in sharp contrast to the case of the Landau-Ginzburg-Wilson theory which only has massive excitations in symmetric phases. Furthermore, we find non-perturbative criteria to characterize quantum phase transitions between competing orders. For example, in 4*D*, we show the two competing order parameter theories, *CP*(1) and the NLSM with WZW, describe different universality class. Physical realizations and experimental implication of the anomalies are also discussed.

Quantum anomaly is one of the most fascinating phenomena in quantum many body systems. Quantum fluctuations spoil classical symmetry, thus corresponding conservation laws and Ward identities no longer hold and must be modified. Topological source terms to the “conservation” laws are induced by quantum fluctuations from anomalies, thus topology and symmetry are intrinsically tied. Consequences of the anomalies were first confirmed in the pion-decay (*π*^0^ → *γγ*), and since then deeper understanding has been achieved^1^,^2^.

Topological protection is one of the most fascinating properties of quantum anomalies, and remarkably this protection is *independent* of interaction strength. ’t Hooft first realized and applied these properties to confinement physics of quantum chromodynamics (QCD), so-called ’t Hooft mathcing, and constrained candidates of low energy degrees of freedom including the Goldstone bosons from the chiral symmetry breaking^3^. Such non-perturbative nature has been extensively applied to high energy physics, for example, the standard model, the Skyrme model of hadrons, and black hole physics^1^,^2^,^3^. In condensed matter systems, it is also applied to several topological phases^4^,^5^,^6^,^7^,^8^,^9^,^10^,^11^,^12^,^13^,^14^,^15^,^16^,^17^,^18^,^19^. For example, the presence of edge states in quantum Hall states and violation of the chiral current conservation in Weyl semimetals are examples of the realizations of the chiral *U*(1) anomaly. Massless excitation in either edge or bulk is protected by anomalies’ topological nature.

In this paper, we consider another realization of quantum anomalies in condensed matter systems, non-abelian anomalies in quantum phase transitions between competing orders and investigate consequences of their presence. We first show that a class of competing order theories has anomalies. Their presence becomes criteria to characterize competing order theories. With the criteria, we find that the competing order theory with the *CP*(1) deconfined transition in three spatial dimensions cannot describe the same universality class of the NLSM with WZW in sharp contrast to the case of two spatial dimensions where the two models are proposed to describe the same universality class. Furthermore, by using the ’t Hooft anomaly matching condition, we also find competing order physics with anomalies *must* contain massless excitation in sharp contrast to the case of the conventional Landau-Ginzburg-Wilson theory. We provide possible candidate theories of the quantum phase transitions with anomalies.

## Theories of Competing Orders

Various order parameters appear in strongly correlated systems, and intriguing interplay between order parameters has been reported^20^,^21^,^22^,^23^,^24^,^25^,^26^,^27^. In this section, we introduce two types of competing order theories, which describes fundamentally different competing order mechanisms, and set up our notation for later discussion. It is worthwhile to mention that we only focus on competing order theories in non-metallic systems in this paper.

**Figure 1:**
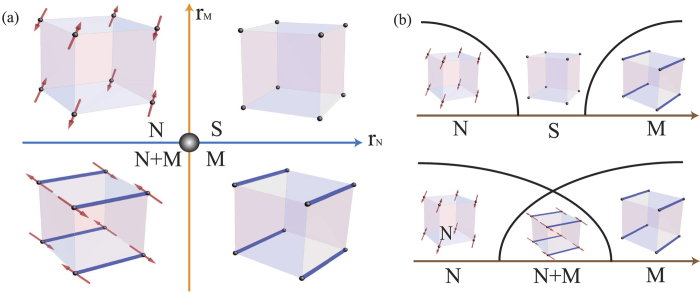
(**a**) Schematic phase diagram of two order parameters in LGW. The horizontal and vertical axes are tuning parameters of the order parameters. Four phases are determined by the signs of (*r*_*N*_, *r*_*M*_): *S* phase (〈*φ*_*N*_〉 = 〈*φ*_*M*_〉 = 0), *N* phase (〈*φ*_*N*_〉 ≠ 0, 〈*φ*_*M*_〉 = 0), *M* phase (〈*φ*_*N*_〉 = 0, 〈*φ*_*M*_〉 ≠ 0), and *N* + *M* phase (〈*φ*_*N*_〉 ≠ 0, 〈*φ*_*M*_〉 ≠ 0). The multi-critical point (0, 0) is well described by a theory with an enlarged symmetry group (*O*(*N* + *M*)). For illustration, we choose *N* = 3 and *M* = 3 (for example, magnetism and valence bond solid) in three spatial dimensions. (**b**) Two generic phase diagrams with one parameter. The vertical axis is for magnitude of order parameters. The upper (lower) one is realized with the condition *r*_*N*_ + *r*_*M*_ > 0(<0), and the fine-tuned condition (*r*_*N*_ + *r*_*M*_ = 0) gives a second order phase transition between the two symmetry broken phases.

The phenomenological *φ*^4^ theory, so-called the Landau-Ginzburg-Wilson (LGW) theory, is one of the simplest ways to describe competing orders. Each order parameter (*φ*_*N*_, or *φ*_*M*_) is a representation of corresponding symmetry group (say, vector representations of *O*(*N*) or *O*(*M*)). The minimal Landau functional is


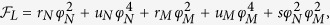


omitting fluctuation terms and higher order terms. Four different phases are basically described by the signs of the tuning parameters (*r*_*N*_, *r*_*M*_) around the multi-critical point (0, 0) as shown in Fig. 1. Note that low energy excitation of the LGW theory’s symmetric ground state (S) is massive which can be easily shown by restoring fluctuation terms. One useful way to understand the phase diagram is to promote the symmetry *O*(*N*) × *O*(*M*) to *O*(*N* + *M*) symmetry by introducing a ‘super-spin’ (*φ*_*N*_, *φ*_*M*_) and focus on the multi-critical point. Then, the four phases are accessed by introducing anisotropy operators. It is well-known based on symmetry that the *φ*^4^ theory is equivalently described by the non-linear sigma model (NLSM)^28^,


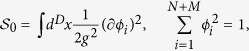


*g* characterizes strength of fluctuations, and the four phases are again accessed by anisotropy operators near the critical coupling constant (*g* = *g*_*c*_).

Another type of competing orders theories can be obtained by incorporating topological terms to NLSMs. It is because NLSMs can easily include topological natures of the compact ground state manifold (*S*^*N*+*M*−1^). There are two types of topological terms in NLSMs: NLSMs with the Wess-Zumino-Witten (WZW) term and NLSMs with the Θ term. The former is realized in *D* = *N* + *M* − 2, which is our main focus in this paper, and the latter is realized in *D* = *N* + *M* − 1, which will be discussed in future work.

The *O*(*D* + 2) NLSMs with the WZW terms in *D* space-time dimensions are


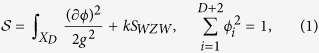


with the spacetime manifold *X*_*D*_. The ground state manifold is *S*^*D*+1^. The WZW term is


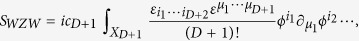


and the presence of the anti-symmetric tensor is related to the winding number of the ground state manifold, 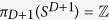. We mainly focus on *D* = 2, 4 though its generalization is straightforward. The boundary of the manifold *X*_*D*+1_ is ∂*X*_*D*+1_ = *X*_*D*_. The numerical constants are 
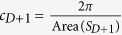
 and 
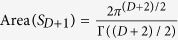
. (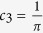 and 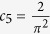). With a non-zero *k*, this class of competing order theories have been suggested to describe exotic competing order physics, for example, competition between Neel and valence bond solid orders associated with deconfined quantum criticality^29^,^30^.

The engineering dimension of the coupling constant is [*g*^2^] = 2 − *D*. In *D* > 2, the coupling constant is irrelevant indicating the model is well-suited to describe a weak coupling fixed point, a symmetry broken phase. Therefore, to access symmetric ground states, one need to consider strong coupling limits where a perturbative calculation is not reliable. Below, we show that non-perturbative nature of quantum anomalies allows us to investigate symmetric phases even in strong coupling limits.

In *D* = 2, the coupling constant is marginal at the tree level, and Witten showed the WZW-NLSM is mapped to a massless fermion model by non-abelian bosonization^31^. This clearly shows that the WZW-NLSM in 2*D* describes a different universality class from the LGW *φ*^4^ theory whose ground state has energy gap as shown by Mermin and Wagner^28^. Thus, it is clear that the WZW term plays a crucial role to modify the ground state in a symmetric phase. Soon after, it was understood that the spin 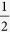 chain is described by the *O*(4) WZW-NLSM, and the onset of the valence-bond-solid order is understood by a marginal perturbation, which is interpreted as competition between spin and valence-bond-solid order. senthil Based on the 2*D* results, the WZW-NLSMs in higher dimensions are suggested to be in a different universality class from the LGW theory applying to various competing order physics^29^,^30^,^32^,^33^,^34^,^35^,^36^,^37^,^38^,^39^,^40^,^41^,^42^,^43^,^44^.

It is, however, significantly more difficult to analyze the models in higher dimensions than 2D partially due to a lack of a local conformal symmetry. Furthermore, a continuous symmetry can be spontaneously broken in higher dimensions, so a symmetry-broken state becomes another stable fixed point, which makes renormalization group flows complicated. The powerful theoretical tools such as *ϵ* = *D* − 2 or large *N* methods are not applicable since changing space-time dimensions and an order parameter manifold are prohibited by the presence of the WZW terms. One conventional way to analyze properties of the symmetric phases is to investigate properties of defects of the order parameters and their proliferation^29^,^30^. This is widely used in literature but its validity is again constrained by a lack of concrete methods. Therefore, it is quintessential to find a concrete way to understand properties of symmetric phases in the WZW-NLSMs. Below, we find one concrete way of the WZW-NLSM type competing order physics in even-spacetime dimensions relying on quantum anomalies.

## Anomalies in WZW-NLSM

We employ the standard strategy to investigate quantum anomalies: to promote a global symmetry to a local one (gauging) and search for inconsistency. weinberg,harvey The promotion is done by introducing a gauge potential and associate minimal coupling. Without the WZW term, the minimal coupling is enough to gauge the symmetry and there is no ambiguity. But, gauging the WZW term is subtle since it is expressed in *X*_*D*+1_. One criterion of a proper gauging procedure is that gauging does not change space-time dimensions of dynamics. For example, equations of motions should be well-defined in the original dimensions (*X*_*D*_) even after gauging since infinitesimally weak gauge coupling is conceivable^45^.

For notation convenience, we use differential forms following Nakahara’s^46^ (also, see Supplementary Materials). In 2*D*, the WZW term is


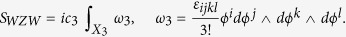


Note that even though the WZW term is expressed in *X*_3_, the equation of motion is well-defined in 2*D*,


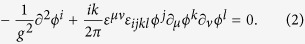


This is because *ω* is the highest form (*dω* = 0) in *X*_3_, and the closeness (*dω* = 0) is tied to a two dimensional equation of motion from Poincare’s lemma. From now on, we focus on *SO*(*D* + 2) symmetry instead of *O*(*D* + 2) since the ’t Hooft anomaly matching only cares continuous groups. The variation of the order parameter in the *SO*(4) vector representation is 
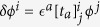
 with the Lie algebra [*t*_*a*_]_*ij*_ = −[*t*_*a*_]_*ji*_ of *SO*(4), 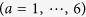. Local dependence of *ϵ*^*a*^(*x*) determines whether the transformation is global or local.

We introduce a gauge potential 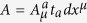 to gauge the *SO*(*D* + 2) symmetry whose field strength is 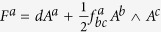. The covarinat derivative is


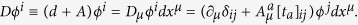


Then, the minimally coupled WZW term in 2*D* is


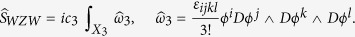


The 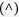 notation is for the minimal coupling with a covariant derivative 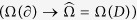. Gauge transformations (*δ*_*g*_) of the potential and the covariant derivative are 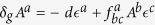 and *δ*_*g*_(*Dϕ*^*i*^) = *ϵ*^*a*^(*x*)[*t*_*a*_]_*ij*_(*Dϕ*^*j*^). By construction, the minimally coupled WZW term is gauge-invariant.

Its equation of motion from the minimal coupling, however, is not two dimensional. This is easily shown by an exterior derivative of the minimally coupled WZW form and we find


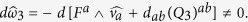


An one-form 
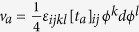
 and the three dimensional Chern-Simon (CS) term, 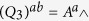 
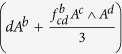
 are introduced. The anomaly coefficient is


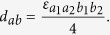


Thus, the simple minimal coupling is not enough to make the WZW term gauge-invariant and the equation of motion two-dimensional.

To make the equation of motion well-defined in 2*D*, one can rearrange and find the closed form. The wedge product (∧) is implicit hereafter.


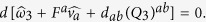


Thus, the gauged total action with a two dimensional equation of motion should be


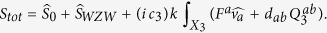


Note that the last two terms contain gauge potential and field strength, so they vanish without gauge fields.

The gauged action, however, has significant inconsistency under gauge transformations


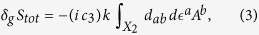


whose origin is the presence of the Chern-Simon (CS) term. Therefore, it is *impossible* to gauge the *SO*(4) symmetry in the WZW-NLSM, which indicates the symmetry is anomalous. Note that similar inconsistency appears at the boundaries of the *U*(1) CS theory in quantum hall systems^4^.

In 4*D* with the *SO*(6) model, the similar procedure is applied with little more tedious calculation. The five dimensional volume form is


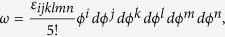


and the exterior derivative of the minimally coupled WZW term is


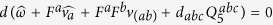


with the anomaly coefficient


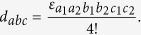


The five dimensional CS term is 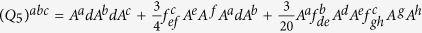, and the three form is 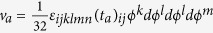. The symmetrized interior derivative is introduced, *i*_(*a*_*v*_*b*)_ = *dv*_(*ab*)_. It is straightforward to show the *SO*(6) symmetry is anomalous.

Note that the anomaly coefficient is only non-zero when all the indices are used up. This indicates that gauging the full *SO*(4) in 2*D* and *SO*(6) in 4*D* is crucial to find anomalies. Gauging subgroups such as *SO*(3) does not use up all the indices, so the coefficient automatically vanishes. In other words, gauging subgroups is always well-defined, and the anomaly structure only appears when the full symmetry is gauged.

Three remarks follows. First, our anomaly coefficient calculation in WZW-NLSMs is *independent* of the coupling constant strength (*g*^2^). This is consistent with non-perturbative nature of quantum anomalies: in the extreme limit with a very large (bare) coupling constant, one can imagine a symmetric ground state. Still, the anomaly structure must be there, so there must be massless excitation to reproduce the anomaly structure since order parameters are completely energy-gapped in symmetric phases. The presence of massless excitation can be more rigorously shown by investigating singularity structure of currents correlation functions^47^,^48^,^49^ (see also Supplementary Materials). Second, the presence of massless excitation in 2*D*, which is consistent with the Witten’s bosonization results, and 4*D* with anomalies indicates their ground states are qualitatively different form LGW theory’s. Thus, our anomaly calculation shows the WZW-NLSMs describes massless symmetric phases qualitatively different from the LGW *φ*^4^ theory and provides non-perturbative criteria to distinguish quantum criticalities. Third, our calculation can also be applied to relations between topological boundary and bulk phases with non-abelian symmetries. The gauge-invariance and equation of motion properties are intrinsically connected through the presence of the *D* + 1 dimensional Chern-Simon terms. Their presence appears in a certain class of D + 1 dimensional topologically ordered phases. Therefore, our work explicitly shows that the D + 1 dimensional (non-abelian) topological phases, related with the non-abelian Chern-Simon theories, can have *D* dimensional gapless boundaries guaranteed by the presence of quantum anomalies.

## Anomaly matching and Minimal model

Quantum anomalies guarantees the presence of massless excitation in symmetric phases but it does not pin down the symmetric ground state completely. But, at least, it is obvious that the conventional strong coupling analysis as in the LGW theory is not applicable to the competing order theories with the WZW term.

A priori, it is not even clear how many phase transitions are and whether they are first or second orders in strong coupling limits. It is because all ignored higher order terms become important in strong coupling limits. Thus symmetric ground states of the WZW-NLSMs are not uniquely determined without further microscopic information. But, no matter what happens, the presence of massless excitation is guaranteed by quantum anomalies.

We first consider one specific model^50^ which reproduces a given quantum anomaly structure which helps us to understand qualitative differences better. Then, we use ’t Hooft matching condition to investigate candidates of symmetric phases. The minimal model to describe a symmetric phase with quantum anomaly can be obtained from the symmetry breaking pattern in the symmetry-broken phases, *SO*(6)/*SO*(5) ~ *S*^5^ in 4*D*. It is well-known in literature that the two color (*N*_*c*_ = 2) QCD with the two flavor (*N*_*f*_ = 2) enjoys the enlarged *SU*(4) flavor symmetry instead of *SU*(2)_*L*_ × *SU*(2)_*R*_^51^,^52^,^53^. The symmetry is spontaneously broken to *Sp*(4) by the chiral condensate. Since *SU*(4) is isomorphic to *SO*(6) and *Sp*(4) to *SO*(5) in terms of the Lie algebra, the symmetry breaking pattern is exactly *SO*(6)/*SO*(5) and the dynamics of the Goldstone boson is described by a *SO*(6) NLSM (see also Supplementary Materials). In 2D, it is well known that spin 1/2 chains realize the *SO*(4) WZW-NLSM.

Inspired by the hints, we construct fermion models coupled to the *SO*(*D* + 2) bosons. We introduce a complex spinor, Ψ, which couples to the order parameters,


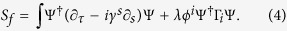


*s* is for spatial dimensions, and *i* is for order parameters. (*γ*^*s*^, Γ_*i*_) matrices satisfy the Clifford algebra. *s* = 1(1, 2, 3), and 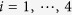 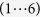 in 2D (4D). By the Yukawa coupling, the fermions become massive in the symmetry broken phases. The minimum numbers of spinor components for the Clifford algebra are four in 2*D* and sixteen in 4*D* as in the spin chain and *N*_*c*_ = 2 QCD.

The WZW term can be easily reproduced in the symmetry broken phases by integrating out the fermions,


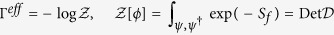


with 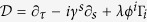. Note that matching the level of the WZW term automatically satisfies the anomaly matching condition, and we indeed find the same level by integrating out the fermions. In Supplementary Materials, standard field theoretic consideration with group structures^1^,^2^ is presented to be self-contained which is useful in symmetric phases. It is also straightforward to show further gradient expansion in the symmetry broken phase of the minimal model reproduces the WZW-NLSM.

The minimal model in 4*D* is naturally constructed by fermions and bosons,


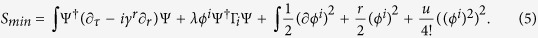


Ψ is in *SO*(6) spinor representation (sixteen complex fields) and *r* is a tuning parameter. In 2*D*, a similar model can be constructed which is well understood, so we focus on 4*D* from now on. By tuning *r*, one can access the symmetry broken phase (*r* < *r*_*c*_) and the symmetric phase (*r* > *r*_*c*_) where *r*_*c*_ is a critical value and its numerical value depends on a renormalization group scheme. The minimal model is a class of the so-called Higgs-Yukawa theory, and uniqueness of the minimal model is the symmetry structure of bosons and fermions associated with quantum anomalies.

**Figure 2:**
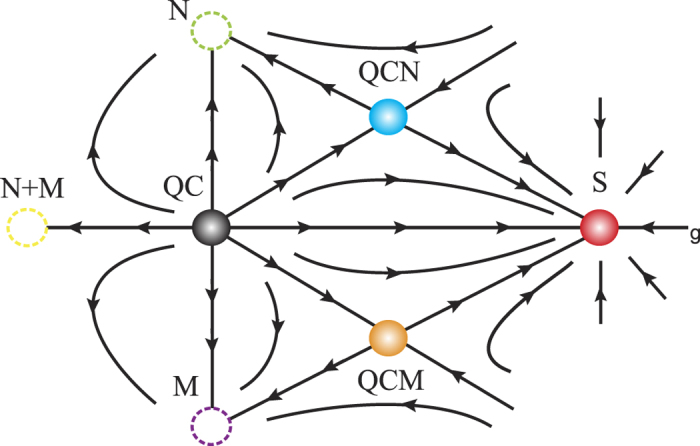
Schematic RG flow of the minimal model in 4D with two parameters. The horizontal (vertical) axis is to characterize fluctuation (anisotropy) strength. *N* + *M* is *SO*(6) symmetry broken phase characterized by 5 Goldstone modes. *M* (*N*) is *SO*(*M*) (*SO*(*N*)) symmetry broken phase characterized by *M* − 1 (*N* − 1) Goldstone modes. *S* is *SO*(6) symmetric phase with massless excitation enforced by quantum anomalies.

The renormalization group flow of the general Higgs-Yukawa theory is well understood^54^, and our minimal model has the same structure. The model is at the upper critical dimension 4*D*, so the mean-field description is valid with logarithmic corrections. There are three fixed points: the symmetry broken phase *N* + *M*, the quantum critical point *QC*, and the symmetric phase *S* (fermionic) as illustrated in the horizontal line of Fig. 2. Note that the symmetry broken phases in the minimal model and the LGW theory are similar, but quantum critical points and symmetric phases are fundamentally different due to the presence of fermions.

We consider perturbations which break the full *SO*(6) symmetry down to its subgroup *H* = *SO*(*N*) × *SO*(*M*), with *N* + *M* = 6 to connect the anomaly structure with competing orders (we treat *SO*(1) ~ **Z**_2_). For example, *SO*(3) × *SO*(3) symmetry in the minimal model allows anisotropy operators


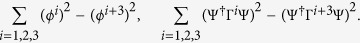


Again, the minimal model is at the upper critical dimension, so one can easily read off scaling dimensions of the operators at each fixed point. Near *QC*, the former operator is relevant because it reduces the number of massless modes but it is irrelevant at *S* since the order parameters are gapped. The four point fermion interaction is irrelevant in all three fixed points. Schematic RG flow of the minimal model with the anisotropy parameter is straightforwardly obtaind as in Fig. 2. Different symmetry breaking patterns with different *N*, *M* give similar RG flows. Note that the RG flow structure of the minimal model is similar to one of the LGW theory, but crucial distinction between the minimal model and the LGW theory is the presence of massless fermions in S enforced by quantum anomalies. If the fermions are identified as electrons, the symmetric phase is nothing but Weyl or Dirac semi-metals^5^.

Before closing this section, we emphasize that the reason we consider the minimal model is to provide a concrete example of the symmetric phases. But, our anomaly calculation is powerful enough to be applied to more generic cases even with strongly correlated ground states. We discuss such more generic cases below.

## Non-minimal Models

Let us consider non-minimal models which have the same anomaly structure. A priori, all conformal field theories (CFTs) with the same anomaly coefficient are candidate theories of S. One straightforward way to construct non-minimal models is to consider different representations of *SU*(4) fermions instead of a single *SU*(4) fundamental representation of the minimal model. Detailed discussion about other representations is presented in Supplementary Materials. Notice that the RG flow structure of the models with different representations is basically the same as the minimal model’s.

If fermions are not electrons but fractionalized particles such as spinons, then the symmetric fixed point can be identified as a spin-liquid phase. If spinons are weakly coupled to gauge field such as *U*(1) or *Z*_2_, then all the properties of the electronic minimal model is inherited and the symmetry broken phases contain remaining gauge structure, so-called * phases. They do not describe conventional symmetry broken phases. Therefore, if the symmetric fixed point with spinons are adjacent to conventional (confined) symmetry broken phases, the gauge structure must be non-abelian. For example, the well-known Banks-Zak fixed point^55^ with spinons could be a candidate of the symmetric fixed point S. Then, condensing the order parameter endows spinon energy gap, and the remaining gauge field becomes confined naturally.

**Figure 3:**
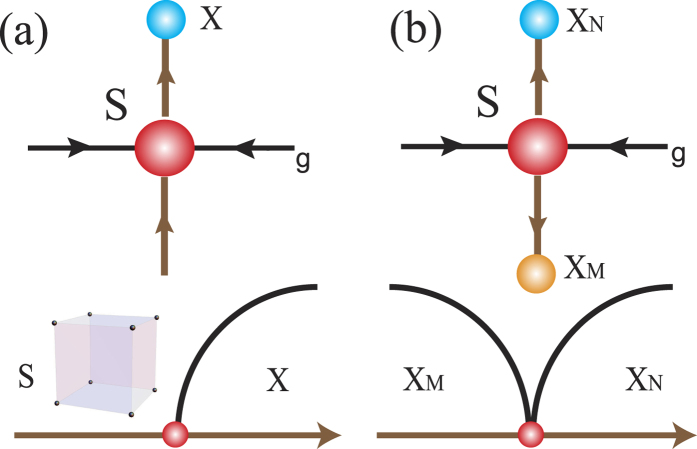
Marginal and relevant operators with *H* = *SO*(*N*) × *SO*(*M*) symmetry. (**a**) RG flow with a marginal operator around the symmetric phase (top). The horizontal (vertical) axis is to characterize fluctuation (anisotropy) strength. Phase diagram with a marginal operator (bottom). Sign of the tuning parameter determines relevance (or irrelevance). Thus, one side of phase diagram is a *SO*(6) symmetric CFT, and the other side (*X*) is either a *H* symmetric CFT or a *H* symmetry broken phase. (**b**) RG flow with a relevant operator around the symmetric phase. The relevant operator drives the RG flow from *S* to *X*_*M*_ and *X*_*N*_. They are either *H* symmetric CFTs or *H* symmetry broken phases. Once *X*_*M*_ and *X*_*N*_ break different symmetries, the symmetric fixed point (*S*) may describe a “deconfined” quantum criticality. The curved (black) lines in the bottom figures are for energy scales associated with symmetry breaking (e.g. order parameter scale).

In principle, two different RG flow structures near S are possible if the symmetric fixed point S is described by strongly coupled CFTs with the same anomalies. In contrast to the minimal model’s RG flow, anisotropy operators could be relevant or marginally relevant. If marginally relevant, then the symmetric phase becomes stable with one definite sign of the coupling constant, but the opposite sign makes the symmetric phase unstable. Thus, one side of phase diagram is described by a *SO*(6) symmetric CFT, and the other side (*X*) is described by either a *H* symmetric CFT or a *H* symmetry broken phase as shown in Fig. 3(a). If relevant, then the symmetric phase becomes unstable with both signs of the coupling constant. Again, the final states can be either *H*-symmetric CFTs or *H*-broken phases as shown in Fig. 3(b). Once the final two states break different symmetries, then the symmetric fixed point (*S*) connects two broken phases directly, which describes deconfined quantum criticality^56^.

The above discussion gives the two necessary conditions to realize deconfined quantum criticality in *SO*(6) WZW-NLSM in 4*D*: anomalies and relevant symmetry breaking operators. These conditions provide non-perturbative criteria to characterize universality class of quantum phase transtions between competing orders. For example, the universality class of the *SO*(6) WZW-NLSM cannot be the same as one of the non-compact *CP*(1) model in 4*D* due to the absence of anomalies in the latter model. Notice that in 3*D* the *CP*(1) model and the *SO*(5) WZW-NLSM are proposed to describe the same universality class but our anomaly criteria do not applied to odd space-time dimensions.

## Discussion and Conclusion

In experiments, direct measurement of the non-abelian anomalies associated with competing orders is even more difficult than one of the chiral *U*(1) anomaly in Weyl semi-metals because we do not know how to couple the non-abelian current directly in experiments^57^,^58^. Yet, there are traits associated with the anomalies.

Protection of massless excitation is one of the most significant characteristics of the presence of quantum anomalies. Their numbers are, however, not universal. For example, in the minimal model in 4*D*, the numbers of massless excitation in Goldstone phase, quantum critical point, and symmetric phase are 5 (bosons), 21(= 6 + 16) (bosons + fermions), and 16 (fermions). In non-minimal models, the symmetry broken phase has the same number, but critical point and symmetric phase have different numbers of massless excitation. Clearly, this is different from LGW theory’s where all massless excitation has definite numbers at each fixed point. The different numbers of massless excitations may contribute to transport differently, which is in principle measurable.

In the minimal model, characteristics of anomaly becomes more evident. First of all, semi-metallic behaviors appear in a symmetry restored phase or high temperature regime (but lower than cut-off scale, say band width) if fermions are electrons. Massless electrons in a symmetric phase (or quantum critical regime) governs low energy physics, so electrical and thermal currents are carried by electrons with the linear spectrum. Naturally, the Wiedemann-Franz law holds especially in non-hydrodyanmic limits. By lowering temperature, the *SU*(4) symmetry can be broken and the electrons become gapped. Thus, electrically, insulating behaviors (energy gap) appear, and the symmetry breaking transition is concomitant with the transition between semi-metal and insulator. On the other hand, Goldstone modes from spontaneous symmetry breaking carry thermal currents even though electrons are gapped. Since Goldstone modes and massless electrons have same dispersion relation, thermal transport in symmetry broken phases is qualitatively similar to the one in symmetric phases. Thus, near the semi-metal and insulator transition, electric and thermal currents behave differently, and the Wiedemann-Franz law would be violated.

One experimentally realizable system of the *SU*(4) anomaly is pyrochlore systems with all-in-all-out magnetic order parameter^59^ in addition to *N*_*c*_ = *N*_*f*_ = 2 QCD. A class of pyrochlore structure is described by a quadratic band touching model^60^ and the onset of all-in-all-out order parameter^61^ induces eight Weyl points (16 fermions), the minimal necessary number to realize the *SO*(6) ~ *SU*(4) anomaly. We note that evidence for all-in all-out ordering and violation of the Wiedemann-Franz law in spin-orbit coupled pyrochlore structures has been reported in literature^62^,^63^ though precise connection with anomalies need further investigation.

In this paper, we investigate non-abelian anomalies in quantum phase transitions with competing orders. We show that the WZW-NLSMs in 2*D* and 4*D* have quantum anomalies by calculating the anomaly coefficients. Non-perturbative nature of the anomalies allows us to investigate not only a symmetry broken phase in weak coupling limit but also a symmetric phase in strong coupling limit even though the presence of the WZW term prohibits conventional *ϵ* = *D* − 2 and large *N* expansion methods. Applying the ’t Hooft matching condition, it is shown that the universality class of the models is qualitatively different from the conventional *φ*^4^ theory’s. In sharp contrast to the *φ*^4^ theory, symmetric ground states of WZW-NLSMs contain massless excitation though their numbers are not uniquely determined. Thus, we construct the minimal model and investigate its properties under anisotropy perturbation. Then, we extend the model to more general ones and discuss implication of the anomalies in competing order physics. Further research on anomalies, for example, parity anomaly in odd space-time dimensions and mixed anomalies with gravity (thermal effects) in connection with topological phases (NLSM with the theta term) are desirable.

## Additional Information

**How to cite this article**: Moon, E.-G. Competing Orders and Anomalies. *Sci. Rep.* **6**, 31051; doi: 10.1038/srep31051 (2016).

## References

[b1] Weinberg S.. The Quantum Theory of Fields. Volume II: Modern Applications. Cambridge University Press (2001).

[b2] Harvey J.. TASI 2003 Lectures on Anomalies. arXiv:hep-th/0509097 (and references therein).

[b3] t Hooft G.. In Recent Developments in Gauge Theories eds t Hooft G. *et al.* 135 (Plenum Press, New York, 1980).

[b4] Wen X. G.. Gapless boundary excitations in the quantum Hall states and in the chiral spin states. Phys. Rev. B 43, 11025 (1991). 10.1103/physrevb.43.11025 9996836

[b5] Vafek O. & Vishwanath A.. Dirac Fermions in Solids: From High-Tc cuprates and Graphene to Topological Insulators and Weyl Semimetals. Annu. Rev. Mater. 5, 83, 67 (2014).

[b6] Ryu S., Moore J. & Ludwig A. W. W.. Electromagnetic and gravitational responses and anomalies in topological insulators and superconductors. Phys. Rev. B 85, 045104 (2012).

[b7] Balents L.. Viewpoint: Weyl electrons kiss. Physics 4, 36 (2011).

[b8] Wen X. G.. Classifying gauge anomalies through symmetry-protected trivial orders and classifying gravitational anomalies through topological orders. Phys. Rev. D 88, 045013 (2013).

[b9] Kapustin A. & Thorngren R.. Anomalous Discrete Symmetries in Three Dimensions and Group Cohomology. Phys. Rev. Lett. 112, 231602 (2014). 24972194 10.1103/PhysRevLett.112.231602

[b10] Wang J., Santos L. H. & Wen X. G.. Bosonic anomalies, induced fractional quantum numbers, and degenerate zero modes: The anomalous edge physics of symmetry-protected topological states. Phys. Rev. B. 91, 195134 (2015).

[b11] Kapustin A.. Symmetry Protected Topological Phases, Anomalies, and Cobordisms: Beyond Group Cohomology. arXiv:1403.1467.

[b12] Sule O., Chen X. & Ryu S.. Symmetry-protected topological phases and orbifolds: Generalized Laughlin’s argument. Phys. Rev. B 88, 075125 (2013).

[b13] Chen X. *et al.* Anomalous Symmetry Fractionalization and Surface Topological Order. Phys. Rev. X 5, 041013 (2015). 10.1103/PhysRevLett.115.236801 26684132

[b14] Else D. & Nayak C.. Classifying symmetry-protected topological phases through the anomalous action of the symmetry on the edge. Phys. Rev. B 90, 235137 (2014).

[b15] You Y. & Xu C.. Topological orders with global gauge anomalies. Phys. Rev. B 92, 054410 (2015).

[b16] Furuya S. & Oshikawa M.. Symmetry protection of critical phases and global anomaly in 1 + 1 dimensions. arXiv:1503.07292. 10.1103/PhysRevLett.118.021601 28128624

[b17] Onkar Parrikar *et al.* Torsion, parity-odd response, and anomalies in topological states. Phys. Rev. D 90, 105004 (2014).

[b18] Can T., Laskin M. & Wiegmann P.. Geometry of Quantum Hall States: Gravitational Anomaly and Kinetic Coefficients. arXiv:1411.3105.

[b19] Gromov A. *et al.* Framing Anomaly in the Effective Theory of the Fractional Quantum Hall Effect. Phys. Rev. Lett. 114, 016805 (2015). 25615495 10.1103/PhysRevLett.114.016805

[b20] Lee P. A., Nagaosa N. & Wen X.-G.. Doping a Mott insulator: Physics of high-temperature superconductivity. Rev. Mod. Phys. 78, 17 (2006).

[b21] Vojta M., Zhang Y. & Sachdev S.. Competing orders and quantum criticality in doped antiferromagnets. Phys. Rev. B 62, 6721 (2000).

[b22] Moon E.-G. & Sachdev S.. Quantum critical point shifts under superconductivity: Pnictides and cuprates. Phy. Rev. B 82, 104516 (2010).

[b23] Hirschfeld P. J., Korshunov M. M. & Mazin I. I.. Gap symmetry and structure of Fe-based superconductors. Rep. Prog. Phys. 74, 124508 (2011).

[b24] Moon E.-G. & Sachdev S.. Competition between spin density wave order and superconductivity in the underdoped cuprates. Phy. Rev. B 80, 035117 (2009).

[b25] Gegenwart P. *et al.* Quantum criticality in heavy-fermion metals. Nat. Phys. 4, 186 (2008).

[b26] Chubukov A.. Pairing Mechanism in Fe-Based Superconductors. Annu. Rev. Cond. Mat. Phys. 3, 57 (2012).

[b27] Moon E.-G. & Sachdev S.. Competition between superconductivity and nematic order: Anisotropy of superconducting coherence length. Phy. Rev. B 85, 184511 (2012).

[b28] Sachdev S.. Quantum Phase Transitions. Cambridge University Press (2nd ed.) (2003).

[b29] Senthil T. & Fisher M. P. A.. Competing orders, nonlinear sigma models, and topological terms in quantum magnets. Phys. Rev. B 74, 064405 (2006).

[b30] Hosur P., Ryu S. & Vishiwanath A.. Chiral topological insulators, superconductors, and other competing orders in three dimensions. Phys. Rev. B 81, 045120 (2010).

[b31] Witten E.., Commun. Non-Abelian Bosonization in two dimensions. Math. Phys. B 92, 455 (1984).

[b32] Tanaka A. & Hu X.. Many-Body Spin Berry Phases Emerging from the -Flux State: Competition between Antiferromagnetism and the Valence-Bond-Solid State. Phys. Rev. Lett. 95, 036402 (2005). 16090760 10.1103/PhysRevLett.95.036402

[b33] Grover T. & Senthil T.. Topological Spin Hall States, Charged Skyrmions, and Superconductivity in Two Dimensions. Phys. Rev. Lett. 100, 156804 (2008). 18518141 10.1103/PhysRevLett.100.156804

[b34] Jaefari A. *et al.* Charge-density wave and superconductor competition in stripe phases of high-temperature superconductors. Phys. rev. B 82, 144531 (2010).

[b35] Fu L. *et al.* Geometric phases and competing orders in two dimensions. Phys. Rev. B 83, 165123 (2011).

[b36] Moon E.-G.. Skyrmions with quadratic band touching fermions: A way to achieve charge 4e superconductivity. Phy. Rev. B 85, 245123 (2012).

[b37] Lu C. K. & Herbut I.. Zero Modes and Charged Skyrmions in Graphene Bilayer. Phys. Rev. Lett. 108, 266402 (2012). 23004998 10.1103/PhysRevLett.108.266402

[b38] Hsu C.-H. & Chakravarty S.. Charge-2 e skyrmion condensate in a hidden-order state. Phys. Rev. B 87, 085114 (2013).

[b39] Goswami P. & Si Q.. Topological defects of Nel order and Kondo singlet formation for the Kondo-Heisenberg model on a honeycomb lattice. Phys. Rev. B 89, 045124 (2014).

[b40] Lee J. & Sachdev S.. Deconfined criticality in bilayer graphene. Phys. Rev. B 90, 195427 (2014).

[b41] Fradkin E. *et al.* Theory of Intertwined Orders in High Temperature Superconductors. Rev. Mod. Phys. 87, 457 (2015).

[b42] Lee J. & Sachdev S.. Wess-Zumino-Witten Terms in Graphene Landau Levels. Phys. Rev. Lett. 114, 226801 (2015). 26196636 10.1103/PhysRevLett.114.226801

[b43] Wang C.. Braiding statistics and classification of two-dimensional charge-2m superconductors. arXiv:1601.02028 (2016).

[b44] Yoni B. & Zee A.. Origin of families and SO18 grand unification. Phys. Rev. D 93, 065036 (2016).

[b45] Hull C. M. & Spence B.. The geometry of the gauged sigma-model with wess-zumino term. Nucl. Phy. B 353, 379 (1991).

[b46] Nakahara M.. Geometry, Topology, and Physics. Taylor and Francis (2nd ed.) (2003).

[b47] Coleman S. & Witten E.. Chiral-Symmetry Breakdown in Large-N Chromodynamics. Phys. Rev. Lett 45, 100 (1980).

[b48] Frishman Y. *et al.* The axial anomaly and the bound-state spectrum in confining theories. Nucl. Phys. B 177, 157 (1981).

[b49] Coleman S. & Grossman B.. ’t Hooft’s consistency condition as a consequence of analyticity and unitarity. Nucl. Phys. B 203, 205 (1982).

[b50] Abanov A. G. & Wiegmann P. B.. Theta-terms in nonlinear sigma-models. Nucl. Phys. B 570, 685 (2000).

[b51] Smilga A. & Verbaarschot J.. Spectral sum rules and finite volume partition function in gauge theories with real and pseudoreal fermions. Phys. Rev. D 51, 829 (1995). 10.1103/physrevd.51.829 10018536

[b52] Kogut J. B., Stephanov M. A. & Toublan D.. On two-color QCD with baryon chemical potential. Phys. Lett. B 464 183 (1999).

[b53] Hill R. J.. SU(3)/SU(2): The simplest Wess-Zumino-Witten term. Phys. Rev. D 81, 065032 (2010).

[b54] Srednicki M.. Quantum Field Theory. Cambridge University Press (2007).

[b55] Banks T. & Zaks A.. On the phase structure of vector-like gauge theories with massless fermions. Nucl. Phys. B 196, 189 (1982).

[b56] Senthil T., Vishwanath A., Balents L., Sachdev S. & Fisher M. P. A.. Deconfined Quantum Critical Points. Science 303, 1490 (2004). 15001771 10.1126/science.1091806

[b57] Hosur P. & Qi X. L.. Recent developments in transport phenomena in Weyl semimetals. Comptes Rendus Physique 14, 857 (2013).

[b58] Burkov A.. Chiral anomaly and transport in Weyl metals. J. Phys.: Condens. Matter 27, 113201 (2015). 25712419 10.1088/0953-8984/27/11/113201

[b59] Witczak-Krempa W. *et al.* Correlated Quantum Phenomena in the Strong Spin-Orbit Regime. Ann. Rev. Cond. Mat. Phys. 5, 57 (2014).

[b60] Moon E.-G. *et al.* Non-Fermi-Liquid and Topological States with Strong Spin-Orbit Coupling. Phys. Rev. Lett. 111, 206401 (2013). 24289698 10.1103/PhysRevLett.111.206401

[b61] Savary L. *et al.* New Type of Quantum Criticality in the Pyrochlore Iridates. Phys. Rev. X 4, 041027 (2014).

[b62] Mandrus D. *et al.* Continuous metal-insulator transition in the pyrochlore Cd2Os2O7. Phys. Rev. B 63, 195104 (2001).

[b63] Sagayama H.. Determination of long-range all-in-all-out ordering of Ir4+ moments in a pyrochlore iridate Eu2Ir2O7 by resonant x-ray diffraction. Phys. Rev. B 87, 100403 (2013).

